# Report of the Committee of Internal Health on the Asiatic Cholera, Together with a Report of the City Physician on the Cholera Hospital

**Published:** 1850-10

**Authors:** 


*Report of the Committee of Internal Health on the Asiatic Cholera, together with a Report of the City Physician on the Cholera Hospital.* Boston. 1849.

This Report is worthy of perusal and preservation. It contains the histories of thirty-three fatal cases of cholera, with the post-mortem appearances. The latter are given with admirable precision, and great minuteness. Appended to the Report are descriptions of the localities in which the disease prevailed, accompanied by daguerreotype views, which convey to the mind of the reader a vivid apprehension of the fact that the special cause of cholera derives its efficiency mainly from filth, want of ventilation, over-crowding, intemperance, and other circumstances appertaining to insalubrity, and neglect of the laws of health.

A map of the city also accompanies the Report, showing the locations in which all the cases at the hospital, and all the fatal cases elsewhere, originated. The course of the epidemic as thus delineated, in Boston, as in other cities, furnishes an almost insuperable difficulty in the way of attributing its diffusion to contagion.

Another fact stated in the Report militates strongly against the doctrine of the personal communicability of the disease. The hospital was situated near the scenes of the greatest ravages of the epidemic. But while several cases originated in its vicinity before it was occupied by patients from other parts of the city, no cases occurred in the houses upon the same square, and the cases which did subsequently occur in the neighborhood were limited to the houses in one direction which were occupied by the most miserable of the population of the city, living in a most miserable manner. Persons residing under better circumstances in the opposite direction from the hospital building, were entirely exempt from the visitation of the malady. These circumstances show that the collection of cholera patients in a hospital did not, in this instance, constitute a focus of contagion; nor can we call to mind any instances that have been reported in which the disease has appeared to spread from cholera hospitals. The conclusions with regard to the various modes of treatment adopted are negative in their character. Patients were treated with narcotics, stimulants, emetics, calomel, quinia, tannic acid, ether, cathartics, venous injection, external heat, the wet sheet, bleeding, etc.; but the comparison of cases in which different remedies were employed does not furnish results upon which a recommendation of any particular plan of treatment can be based. In the great majority of instances patients were admitted in a state of complete collapse, and the large ratio of fatality under different methods of treatment, go to confirm, what we fear must be admitted as a truth, that under such circumstances active remedies are seldom successful, and perhaps not infrequently injurious. In its incipient stage cholera is perfectly manageable, and requires only simple remedies, with proper hygienic regulations; but after this favorable period has passed, the question of life and death turns on the degree of the blood lesions that have occurred, and the amount of the recuperative energies of the organism. Perturbating treatment under the latter circumstances is, to say the least, questionable.

The reporter states that an invitation was extended to several homœopathic practitioners to test the vaunted efficacy of infinitesimal doses in the treatment of the disease. None, however, were willing to come into the hospital upon equal terms with the city Physician, and take charge of an equal number of patients.

The remarks on the pathology of the disease, based on the autopsical histories, are sensible, but the interest of the subject, at this time, is perhaps not sufficient to devote to it space enough to do justice to the views presented in this portion of the report. There is one point, however, to which we will briefly advert, inasmuch as the ideas generally entertained respecting it, exert, with many practitioners, a controlling influence in the election of remedies. We refer to the biliary secretion. It has been generally supposed that an attack of cholera is uniformly attended by suppression of bile. On reference to the autopsies contained in this Report, it will be found that bile was frequently found after death in the duodenum and stomach, almost always being present in the gall-bladder, and generally expressed without difficulty from the small ducts in the interior of the liver. The theory which refers the disease to congestion of the portal circle, dependent on suppression of the bile, appears to us wholly inadequate to account for the phenomena, assuming the facts to be precisely as they are taken for granted. But it would seem from the observations contained in this Report, that the liver is even less affected than other glands—certainly less than the renal organs. The supposed efficacy of calomel in this disease is rationally explained by its supposed action on the liver. In view of the facts just referred to, some other explanation must be sought for. That the bilious stools, as they are termed, occur in cases in which mercurials have not been given, our own observations have afforded abundant evidence.

In concluding this notice of this able and truly valuable Report, we should not omit to state that it was submitted to the Mayor and aldermen of Boston, by Dr. Henry G. Clark, city Physician, having been prepared by him in conjunction with Dr. Charles E. Buckingham, John C. Dalton, and Henry W. Williams.

